# Mechanically-evoked C-fiber activity in painful alcohol and AIDS therapy neuropathy in the rat

**DOI:** 10.1186/1744-8069-3-5

**Published:** 2007-02-23

**Authors:** Xiaojie Chen, Jon D Levine

**Affiliations:** 1 Departments of Anatomy, Medicine and Oral and Maxillofacial Surgery, Division of Neuroscience, NIH Pain Center, University of California, San Francisco, CA 94143, USA

## Abstract

While altered activities in sensory neurons were noticed in neuropathic pain, caused by highly diverse insults to the peripheral nervous system, such as diabetes, alcohol ingestion, cancer chemotherapy and drugs used to treat AIDS, other infections and autoimmune diseases, as well as trauma, our understanding of how these various peripheral neuropathies manifest as altered neuronal activity is still rudimentary. The recent development of models of several of those neuropathies has, however, now made it possible to address their impact on primary afferent nociceptor function. We compared changes in mechanically-evoked C-fiber activity, in models of painful peripheral neuropathy induced by drinking ethanol (alcohol) or administering 2',3'-dideoxycytidine (ddC), a nucleoside reverse transcriptase inhibitor for AIDS therapy, two co-morbid conditions in which pain is thought to be mediated by different second messenger signaling pathways. In C-fiber afferents, ddC decreased conduction velocity. In contrast, alcohol but not ddC caused enhanced response to mechanical stimulation (i.e., decrease in threshold and increase in response to sustained threshold and supra-threshold stimulation) and changes in pattern of evoked activity (interspike interval and action potential variability analyses). These marked differences in primary afferent nociceptor function, in two different forms of neuropathy that produce mechanical hyperalgesia of similar magnitude, suggest that optimal treatment of neuropathic pain may differ depending on the nature of the causative insult to the peripheral nervous system, and emphasize the value of studying co-morbid conditions that produce painful peripheral neuropathy by different mechanisms.

## Background

The second messenger signaling pathways in primary afferent nociceptors that mediate hypersensitivity to mechanical stimuli differ between models of painful peripheral neuropathies [1]. Two extreme examples of this are the neuropathies induced by chronic ethanol consumption, and by acquired immunodeficiency disease syndrome (AIDS) therapy (nucleoside reverse transcriptase inhibitors). In alcohol-induced neuropathy, protein kinase Cε(PKCε) has a major contribution to mechanical hyperalgesia [2], whereas in AIDS therapy neuropathy, Ca^++^, caspase signaling and mitochondrial electron transport [3-5] but not PKCε or a number of other second messenger signaling pathways (i.e., protein kinase A, protein kinase G, extracellular signal-regulated kinases 1/2 or nitric oxide) contribute [3].

Enhanced activity in sensory neurons is thought to contribute to pain reported by patients with small-fiber peripheral neuropathies. Microneurography techniques have demonstrated pathological responses such as sensitization to mechanical stimuli, in patients with trigeminal neuralgia [6], traumatic nerve injury [7], entrapment neuropathy [8], phantom limb [9] and erythromelalgia [10]. However, there are practical limitations in performing microneurography in patients, including inability to classify fiber functions fully, small numbers of fibers that can be evaluated in an individual patient and the potential for inducing further injury by introducing a microelectrode into an already damaged nerve. Furthermore, in spite of the fact that in most patients, metabolic abnormalities, toxins, drugs or infectious organisms are producing the neuropathic conditions, most microneurography studies have been done in patients with a traumatic nerve injury [7-9].

Single-fiber electrophysiology has been performed in animal models of metabolic and toxic, as well as traumatic nerve injury-associated painful peripheral neuropathy. Following traumatic nerve injury it has been reported that there is increased spontaneous activity occurring in irregular bursts [11-13]; in diabetic neuropathy, in addition to increased spontaneous activity, a decrease in threshold and increase in response to supra-threshold stimulation has been reported [14-19]; in models of cancer chemotherapy neuropathy, C-fibers have been reported to be hyperresponsive and to fire irregularly [1,20]; in alcohol neuropathy, C-fibers also demonstrate a decrease in threshold and increased response to stimulation [2]; and, in nucleoside reverse transcriptase inhibitor-induced AIDS-therapy neuropathy, a change in post-stimulus interspike interval (ISI) histogram, without change in threshold or number of action potentials in response to threshold or suprathreshold mechanical stimulus has been reported [3]. In this study, we have performed a side-by-side comparison of evoked C-fiber activity in models of two frequently co-morbid forms of peripheral neuropathy, alcohol and AIDS therapy-induced painful peripheral neuropathy, which differ markedly in the nociceptor second messenger signaling pathways involved [2,3].

## Results

### Conduction velocity

**Figure 1:**
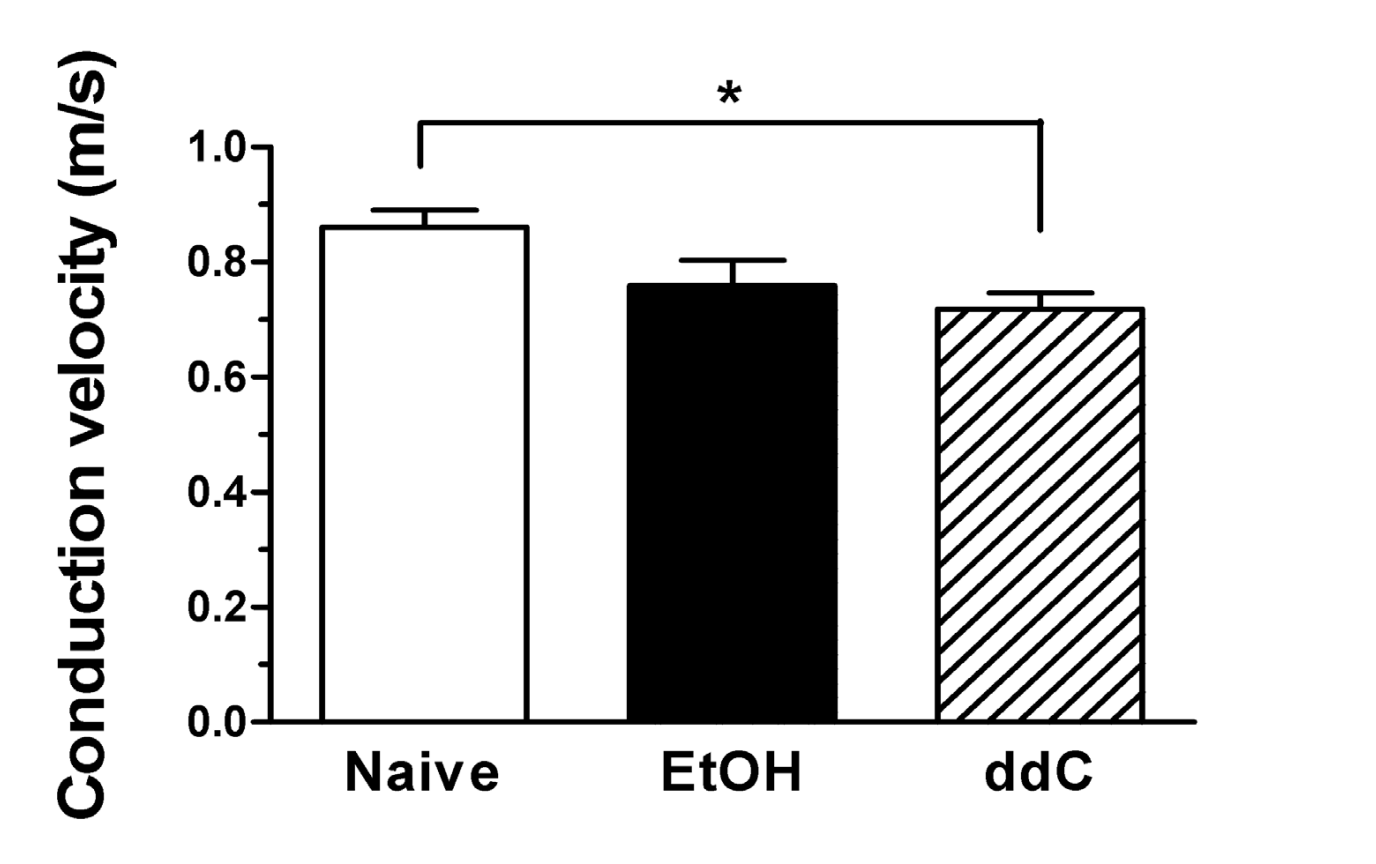
Mean conduction velocities (0.86 ± 0.03, 0.76 ± 0.04, 0.72 ± 0.03 m/sec) of C-fibers from the three groups of rats (i.e., naive, ethanol (EtOH) and ddC, respectively) were significantly different (one way ANOVA, p < 0.05). The conduction velocity of C-fibers from the ddC group (n = 18) was significantly lower than that of control rats (n = 38, p < 0.05) while the conduction velocity was similar between EtOH (n = 15) and control groups (p > 0.05).

Conduction velocity, a measure of axonal excitability, has been used extensively in the classification and diagnosis of peripheral neuropathies. The conduction velocity of individual C-fibers, whose mechanical receptive fields had been identified, was measured in sensory neurons innervating the dorsum of the hind paw of ethanol-consuming and ddC-treated rats that demonstrated mechanical hyperalgesia prior to electrophysiology study, and in control rats. While there was a decrease in conduction velocity in both ethanol (decrease 11.7%) and ddC (decrease 16.4%) treated rats, the decrease was statistically significant only in the AIDS therapy model (Figure 1, p < 0.05). Thus, as in patients with diverse forms of peripheral neuropathy who have a decrease in conduction velocity in *myelinated *fibers, a decrease in the conduction velocity in C-fibers of rats with peripheral neuropathy can also be shown. Since it is generally considered that slowed conduction velocity is a manifestation of alterations in axonal ionic conductance [21], our findings are compatible with changes in ionic conductance in C-fiber axons in AIDS therapy neuropathy. How such changes might contribute to symptoms associated with ddC peripheral neuropathy requires further studies.

### Response to mechanical stimulation

#### Threshold stimulus intensity

**Figure 2:**
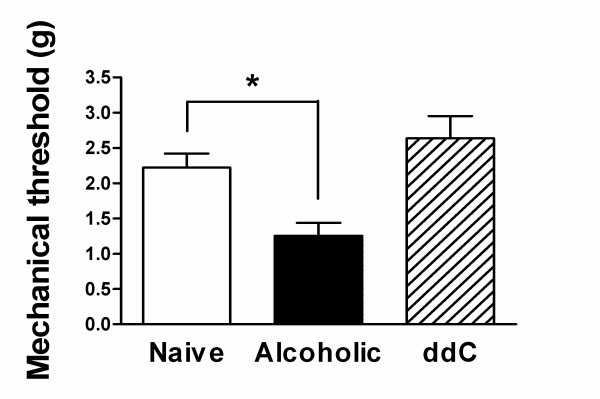
The mechanical threshold of C-fibers in the EtOH group (n = 15) was significantly lower that of control C-fibers (n = 38, p < 0.05, Mann Whitney test) while the mechanical thresholds between ddC (n = 18) and control groups were similar (p > 0.05, Mann Whitney test).

Alterations in primary afferent nociceptor function associated with enhanced pain are thought to be due to a decrease in threshold for nociceptor activation and an increase in number of action potentials fired. Therefore, the mechanical threshold and response at threshold of C-fiber nociceptors was determined in control as well as in alcohol-consuming and ddC-treated rats. In contrast to conduction velocity, changes in most of the other electrophysiological parameters occurred only in the rat model of alcohol-induced painful peripheral neuropathy. Thus, chronic ethanol ingestion but not ddC administration produces a decrease in average C-fiber mechanical threshold (43.5%; p < 0.05); in ddC-treated rats there was actually a small, not statistically significant, *increase *in mechanical threshold (Figure 2).

#### Response to sustained threshold and suprathreshold stimulus

**Figure 3:**
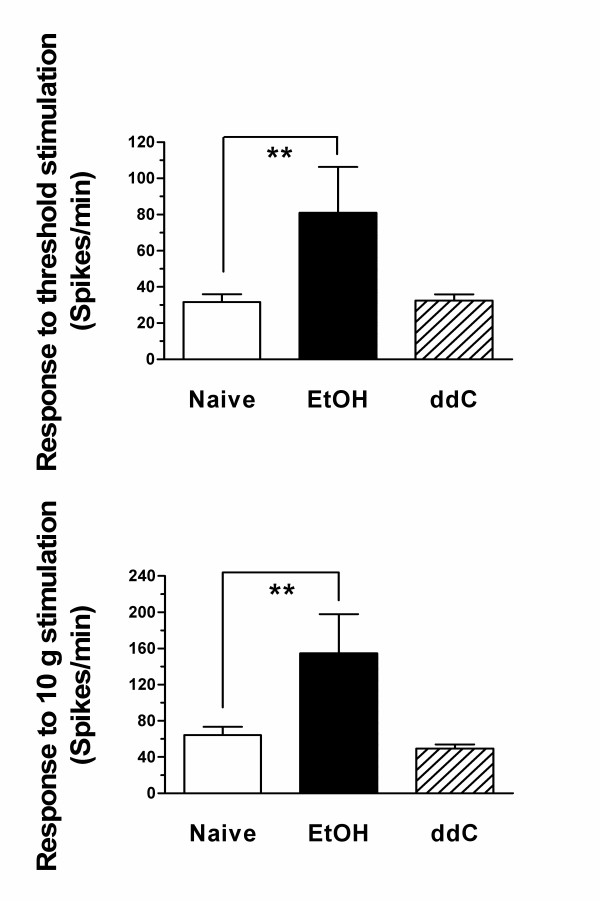
The mean responses to both sustained (60 sec) threshold and 10 g stimuli of ddC, EtOH and control C-fibers were significantly different (one way ANOVA, p < 0.01). The responses of EtOH group (n = 15) were significantly higher than those of control rats (n = 38, p < 0.01, Tukey's multiple comparison test) while the responses of C-fibers in the ddC group (n = 18) were similar to those of controls (p > 0.05, Tukey's multiple comparison test).

Similar to their effects on C-fiber mechanical threshold, ethanol consumption but not ddC administration significantly enhanced the number of action potentials fired in response to sustained threshold and fixed suprathreshold (10 g) intensity mechanical stimulation (Figure 3). These results provide further support for the suggestion that enhanced C-fiber response contributes to the symptoms of alcohol-induced peripheral neuropathy and raises the question of how the enhanced nociception in AIDS therapy painful peripheral neuropathy is encoded. Thus, while our data support a role for enhanced C-fiber response contributing to pain associated with alcohol neuropathy, it does not appear to contribute in AIDS therapy neuropathy.

### Activity pattern

#### ISI

**Figure 4:**
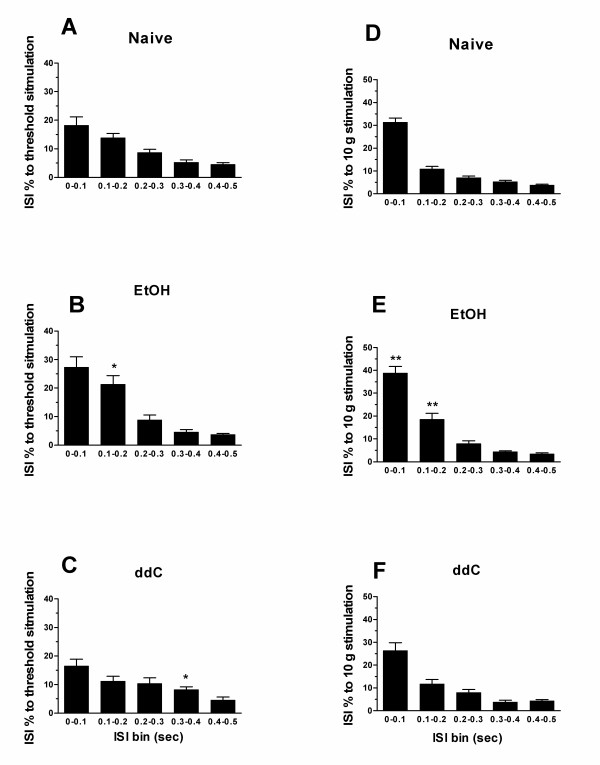
The ISI distributions of both EtOH (n = 15) group of C-fibers in responses to sustained (60 sec) threshold and 10 g stimuli were significantly changed. **A&D**, the ISI distributions of control C-fibers (n = 38) in responses to sustained threshold and 10 g stimuli, respectively. **B&C**, ISI 0.1–0.2 s of responses in EtOH group was significantly higher than that of controls (p < 0.05, t-test) and ISI 0.3–0.4 s of responses in ddC group (n = 18) was significantly higher than that of controls (p < 0.05, t-test). **E&F**, the ISI distributions of EtOH group in responses to 10 g stimulation were significantly changed while the ISI distributions of ddC group were similar to those of controls. ISI 0–0.2 s of responses in EtOH group was significantly higher than those of control animals (p < 0.01, t-test)

In spite of the ability of activity pattern to signal, independent of average firing frequency [20,22], changes in activity pattern produced by various forms of painful peripheral neuropathy has not been studied systematically. To analyze the changes in activity pattern generated in response to stimulation of C-fiber nociceptors in the mechanical receptive field, we first analyzed the ISI histograms for the response of C-fibers to sustained (60 sec) threshold and suprathreshold (10 g) mechanical stimulation, in ethanol-consuming, ddC-treated and control rats. In alcohol consuming rats, the proportion of short ISIs was significantly increased (p < 0.05, Figure 4B; p < 0.01, Figure 4E). A small, albeit statistically significant, increase in intermediate ISI was observed with threshold stimulus, in ddC-treated rats (Figure 4C). That there is an increase in the number of short ISIs in rats with alcohol neuropathy, suggests that temporal summation may play a role in the neuropathic symptoms associated with alcohol consumption but not ddC treatment.

#### Co-efficient of variability (Cv2)

**Figure 5:**
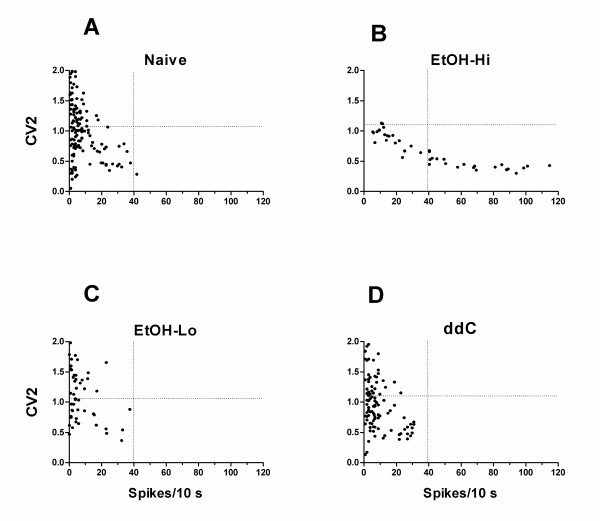
The Cv2 distribution of high firing fibers (**B**, n = 7) for EtOH group is markedly different from low firing fibers (**C**, n = 8) for EtOH group and control animals (**A**, n = 38) while they were similar between low firing fibers for EtOH group and controls. The Cv2 distributions were similar between ddC (**D**, n = 18) and controls.

Finally, changes in activity pattern, generated in response to mechanical stimuli, was analyzed by determining the coefficient of variability (Cv2) distribution for the response to sustained threshold and suprathreshold mechanical stimulation in C-fibers from ethanol fed, ddC treated or control rats. The plots of Cv2 versus number of spikes, in response to mechanical stimulation, for high-firing fibers in alcohol fed rats were different from that of low-firing and control fibers (Figure 5). In these high-firing fibers, the maximum Cv2 values were less, such that there were almost no occurrences of Cv2 values greater than 1.1, unlike the distribution of Cv2 values in low-firing and control C-fibers. The variability of Cv2 values was also smaller. These changes contrast with those observed in vincristine and diabetic neuropathy [16,20], where high and variable Cv2 values were observed in high-firing fibers. In ddC-treated rats there were no hi-firing C-fibers; the Cv2 distribution for C-fibers in ddC-treated rats was similar to that for C-fibers from controls rats (Figure 5A&D).

## Discussion

While it is generally accepted that enhanced activity in primary afferent nociceptors plays an important role in the pain experienced by patients with peripheral neuropathy [6-10], changes in activity in primary afferent nociceptors have received little attention, including direct comparisons between changes in primary afferent nociceptor function in different forms of painful peripheral neuropathy. In this study, we have compared mechanically evoked C-fiber activity in rat models of alcohol and AIDS therapy-induced peripheral neuropathy, for which enhanced nociception has been shown to be dependent on different second messenger signaling pathways.

While the mechanical hyperalgesia observed in these two models of painful peripheral neuropathy are of similar magnitude [2,3], the changes in C-fiber function differ markedly, being fairly well restricted to a decrease in conduction velocity for AIDS therapy, while many aspects of mechanically-evoked activity were effected by alcohol. Although clinical studies show slowed conduction velocity in many types of peripheral neuropathy [21,23-25], the mechanism underlying slowing of conduction velocity remains to be established. Available data suggest that changes in ionic currents, most especially for voltage-gated ion channels, contribute to conduction velocity abnormalities [21,26,27]. The most well studied model with respect to mechanisms involved in changes in conduction velocity is diabetic neuropathy [21], in which I_Na+_, I_K+ _and I_h _have been shown to decrease [26,27] and I_Ca2+ _to increase [28-30]. However, depending on the composition of other ion channels in the membrane of the sensory neuron, one may observe either enhanced or attenuated sensation [21]. Since slowing of nerve conduction velocity is the major change in C-fiber function in the rat model of ddC-induced painful peripheral neuropathy, direct analysis of ionic currents in dorsal root ganglion neurons treated with AIDS therapy could provide important insights into the mechanisms involved in the pain associated with this class of neuropathies.

While the relatively small change in single fiber electrophysiological properties of primary afferent nociceptors observed in rats with ddC neuropathy might suggest that changes in the peripheral terminal of sensory neurons make a minor contribution to AIDS therapy-induced pain, we have previously shown that peripheral administration, at the site of nociceptive testing, of antagonists of intracellular calcium [3], caspase signaling [4] and the mitochondrial electron transport chain [5], which in control animals have no effect on mechanical nociceptive threshold, reverses ddC-induced mechanical hyperalgesia. Taken together these findings provide support for the suggestion that changes in primary afferent nociceptor function, not tested for in the present study, may play a role in the decreased behavioral mechanical nociceptive threshold in the ddC-induced painful peripheral neuropathy. Alternatively, since the mechanism of action of nucleoside reverse transcriptase inhibitor-induced neurotoxicity is via their effects on mitochondrial function [31,32], it may be that a fraction of mitochondria are affected in most neurons, leading to a smaller change in function in a larger percentage of sensory neurons. In contrast, in alcohol, diabetic [16,17] and vincristine [20] peripheral neuropathy, the toxic insult appears to produce an all-or-none change in activity, in a subset of neurons (i.e., the high-firing fibers) not observed in AIDS therapy neuropathy.

Decrease in mechanical threshold and increase in number of action potentials elicited by the same intensity stimulus contribute to inflammatory pain [33,34], which is characterized by mechanical hyperalgesia. In the present study we found a decrease in mechanical threshold and increase in number of action potentials produced by threshold and suprathreshold stimulation in rats consuming alcohol, but not in ddC-treated rats. The increase in number of short ISIs, in response to both threshold and suprathreshold mechanical stimulation, in alcohol fed rats, will increase temporal summation in postsynaptic spinal dorsal horn neurons; increasing the range of ISIs, near 100 ms, as observed in rats consuming alcohol, causes greater temporal summation of C-fiber-evoked excitatory postsynaptic currents in dorsal horn neurons [35], and in the same range of ISIs, temporal summation of afferent activity appears to be an important factor in human pain perception [36-41].

While pattern of activity in a presynaptic neuron can dramatically affect activity in its postsynaptic neurons [42-46], much less attention has been given to the importance of the pattern of activity elicited by mechanical stimulation of primary afferents in the pain associated with peripheral neuropathy. In previous studies of painful peripheral neuropathy we have observed that changes in primary afferent nociceptor function occur in an all-or-nothing fashion. Thus, in models of diabetic [16,17] and vincristine [20] neuropathy, we found enhanced activity restricted to a subpopulation of C-fibers (i.e., high-firing fibers), the function of the remaining C-fibers being similar to those in control rats. In alcohol-induced neuropathy this dichotomy was also present. Therefore, in our analysis of variability in action potential timing we also separately evaluated the change in activity pattern in high- and low-firing C-fibers. Marked alteration in the distribution of Cv2 values was observed in high-firing C-fibers in alcohol-induced painful peripheral neuropathy; however, this change was different from that in high-firing C-fibers in diabetic and vincristine-treated rats, in that there was a marked decrease in maximum Cv2 in rats with alcohol-induced neuropathy. While the mechanism underlying these changes is unknown, the lower Cv2 value can be generated by a repetitively bursting pattern of activity [42]. The functional significance of variability in neuronal discharge patterns has been the focus of study in somatosensory cortex and other sensory areas [42-44,46]. It has been suggested that such "variability may not be so much a flaw as a feature that the brain puts to good use" to "provide the dynamic range for rapid modulation of synaptic efficacy" [45]. This may be relevant to the function of nociceptors as afferent activity-dependent plasticity in spinal nociceptive pathways is thought to be a crucial feature of pain signaling [47], and may contribute to the progressive increase in pain during a prolonged stimulus, even while adaptation decreases the mean firing frequency of nociceptive nerve fibers [48].

In summary, in two models of painful peripheral neuropathies that differ markedly based on the involvement of second messenger signaling mechanisms in primary afferents, we have found marked differences in C-fiber activity. Our findings raise the question; does activity in sensory neurons from different forms of peripheral neuropathy have unique signatures? Since alcohol consumption and AIDS are common co-morbid conditions [49-51], the possibility that they produce painful peripheral neuropathy by different mechanisms raises the question are symptoms more severe in AIDS patients who chronically consume alcohol? One step in developing an understanding of the importance of these mechanisms would be to directly activate individual second messengers in primary afferent nociceptors, to determine their effect on mechanically-evoked nociceptor activity, and then to study specific ion channels in dorsal root ganglion neurons, *in vitro*, to determine the ionic basis of these differences. *In vitro *studies of the effect of ddC on specific ionic conductance may be especially important in furthering our understanding of the functional alterations in AIDS therapy neuropathy, which does not appear to markedly alter function of individual primary nociceptors.

## Conclusion

Our results demonstrated that only ddC decreased conduction velocity of C-fiber afferents. In contrast, alcohol but not ddC caused enhanced response to mechanical stimulation and changes in pattern of evoked activity. Our data also support the suggestion that different therapies are likely to be needed to effectively manage symptoms in different forms of peripheral neuropathy.

## Methods

### Animal model

Male Sprague-Dawley Rats (280–420 g) from Bantin and Kingman (Fremont, CA, USA) were used in these experiments. Animal care and use conformed to National Institutes of Health (NIH) guidelines and was approved by the University of California at San Francisco Committee on Animal Research.

### Alcohol-induced painful neuropathy

The rats used in these experiments housed one per cage were fed Lieber-DeCarli liquid diet (Dyets Inc., Bethlem, PA) containing ethanol (6.5% ethanol) [2,52-54] for 12 weeks. In this protocol, alcohol-induced hyperalgesia is well established by the end of the seventh week and maximal between 8–12 weeks [2]. All rats demonstrated mechanical hyperalgesia prior to electrophysiology study.

### 2',3'-dideoxycytidine-induced neuropathy

The nucleoside reverse transcriptase inhibitor for AIDS therapies induces a painful peripheral neuropathy in the rat [3]. A single dose of the AIDS therapy drug, 2',3'-dideoxycytidine (ddC, 50 mg/kg i.v.), produces a significant reduction in nociceptive threshold from day 1 after its administration, which persisted for more than 20 days [3]. Since the model produced mechanical hyperalgesia in 100% of animals [3], we did not perform behavioral studies for each of the animals used in the electrophysiology experiments. Of note, this would only have the potential to underestimate the effect of neuropathy on sensory neuron function.

### Electrophysiology

*In vivo *single-fiber electrophysiology was performed, as previously described [16,18,20]. Briefly, rats were anesthetized with sodium pentobarbital (initially 50 mg/kg, i.p., with additional doses given throughout the experiment to maintain areflexia). At the end of the experiment the rat was euthanized by pentobarbital overdose followed by bilateral thoracotomy. Recordings were made from the saphenous nerve, which innervates the dorsal surface of the hind paw. Bipolar stimulating electrodes were placed under the nerve at a site distal to the recording site. The nerve was crushed proximal to the recording site to prevent flexor reflexes during electrical stimulation of the nerve. Fine fascicles of axons were dissected from the nerve and placed on a silver-wire recording electrode. Single units were first detected by electrical stimulation of the nerve. Receptive fields of identified C-fibers were located using a mechanical search stimulus, either a blunt probe with smooth tip or a 60 g von Frey hair (VFH). Each fiber's conduction velocity was calculated by dividing the distance between the stimulating and recording electrodes by the latency of the electrically-evoked action potential. Fibers that conducted slower than 2 m/s were classified as C-fibers [55,56]. The fiber was determined to be cutaneous if it was activated by lifting and stimulating the skin and/or by moving skin with respect to its subcutaneous tissue. All C-fibers employed in the present experiment had cutaneous receptive fields. The electrically evoked action potential corresponding to the C-fiber whose receptive field had been identified was verified by the latency delay technique, in which electrically evoked spikes resulted in longer latency when the receptive field of the same fiber was stimulated mechanically [57]. Mechanical threshold was determined with calibrated VFH and defined as the lowest force that elicited ≥2 spikes within 1 s, in at least 50% of trials.

Sustained threshold mechanical stimulation was performed using a calibrated VFH that was manually placed on the receptive field for 60 s. Sustained (60 s) suprathreshold (10 g) mechanical stimulation was accomplished by use of a mechanical stimulator consisting of a force-measuring transducer (Entran, Fairfield, NJ, USA) mounted in series with interchangeable VFH filaments. Neural activity was stored using an IBM compatible computer with micro 1401 interface (CED, Cambridge, UK) and further analyzed off-line with Spike2 software (CED).

### ISI analysis

ISI analysis was used to evaluate the temporal characteristics of the response of C-fiber nociceptors to sustained mechanical stimulation, which was adopted from our study of nociceptor activity in the rat model of vincristine-induced painful neuropathy [20]. The ISIs for each C-fiber's response was grouped into 100 ms bins between 0 and 499 ms; ISIs greater than or equal to 500 ms were not analyzed [20]. This bin width also allows our data to be compared with that in other studies [35,58,59]. The number of intervals occurring in each bin was expressed as the percentage of the total number of ISIs in the trial. This trial-by-trial normalization procedure allowed the distribution of ISIs from several fibers to be averaged together.

### Action potential firing variability (Cv2)

The coefficient of variability (Cv2) was calculated to compare the relative difference between adjacent ISIs [42]. Cv2 is defined as the square root of 2 multiplied by the standard deviation of two ISIs divided by their mean [42]. Thus, it is a dimensionless value that is independent of absolute firing rate. Based on our previous studies in rat models of vincristine- and diabetes-induced peripheral neuropathy [16,20], the response of each C-fiber during the 1 min duration of the stimulus was divided into six consecutive 10 s periods, and the average Cv2 for all fibers in each corresponding 10 s period was calculated. Based on our similar finding in rat models of vincristine and diabetes-induced painful peripheral neuropathy [16,20], fibers were divided into two groups, "low-firing" fibers which fired <100 spikes and "high-firing" fibers which fired >100 spikes, so that the firing pattern of C-fiber activity from the peripheral neuropathy models can be compared. The high-firing C-fibers had approximately 2.5-fold higher responses to sustained threshold and suprathreshold mechanical stimulation compared with control fibers during the 60 s stimulus while the low-firing frequency C-fibers had responses similar to those of controls.

### Statistics

Group data are expressed as mean ± S.E.M. Statistical analyses were done using analysis of variance (ANOVA) followed by Tukey's multiple comparison post hoc test or unpaired *t*-test and Mann Whitney *U *test, as appropriate. Differences were considered significant at *P *< 0.05.

## List of abbreviations

ddC : 2',3'-dideoxycytidine

ISI : interspike interval

AIDS : Acquired Immunodeficiency Disease Syndrome

PKCε : protein kinase Cε

VFH : von Frey hair

Cv2 : coefficient of variability

EtOH : ethanol

## Authors' contributions

XC participated in the design of the study, carried out all the experiment, performed the statistical analysis and drafted the manuscript. JDL participated in the design of the study and drafted the manuscript. All authors read and approved the final manuscript.
